# Resultados da artroplastia total do joelho com assistência robótica

**DOI:** 10.1055/s-0045-1814407

**Published:** 2025-12-30

**Authors:** Joao Paulo Fernandes Guerreiro, Julia Canassa Pinto, Livia Schauff, Tiago Delfino Pedrollo, Paulo Roberto Bignardi, Marcus Vinicius Danieli

**Affiliations:** 1 Pontifícia Universidade Católica do Paraná, Londrina, PR, Brasil; 2 Hospital de Ortopedia Uniorte, Londrina, PR, Brasil

**Keywords:** artroplastia, joelho, perda sanguínea cirúrgica, procedimentos cirúrgicos robóticos, arthroplasty, blood loss, surgical, knee, robotic surgical procedures

## Abstract

**Objetivo:**

Investigar os resultados da artroplastia total do joelho (ATJ) com assistência de braço robótico em comparação com a abordagem convencional.

**Métodos:**

Realizamos um estudo de coorte retrospectivo, incluindo 96 pacientes submetidos a ATJ, que foram alocados aos grupos assistência robótica (AR) ou técnica convencional (TC). Todas as cirurgias foram realizadas sem o uso de torniquete e incluíram a administração de ácido tranexâmico intravenoso. Os pacientes foram pareados com base no sexo, na idade e nos valores pré-operatórios de hemoglobina (Hb) e hematócrito (Ht). Os principais desfechos avaliados foram a perda sanguínea perioperatória (avaliada por meio de alterações em Hb e Ht), a duração operatória, o tempo de internação hospitalar e as complicações pós-operatórias até em 6 meses.

**Resultados:**

Foram 34 pacientes para cada grupo pareados com sucesso para análise. Não houve diferenças significativas entre os grupos quanto à redução de Hb ou Ht (AR: Hb −2,27 ± 1,21 g/dL, Ht −6,56 ± 3,43%; vs. TC: Hb −2,00 ± 1,07 g/dL, Ht −5,85 ± 3,26%; *p* > 0,05). O tempo cirúrgico médio também foi semelhante (AR: 108,9 ± 20,8 vs. TC: 111,8 ± 26,2 min; *p* = 0,905). Notavelmente, os pacientes do grupo AR tiveram um período de hospitalização mais curto (mediana: 2 vs. 2,5 dias; OR = 0,12; IC 95% = 0,03–0,57; *p* = 0,008). As complicações pós-operatórias em 6 meses não diferiram significativamente entre os grupos.

**Conclusão:**

A ATJ com assistência robótica não foi associada a melhorias mensuráveis na perda sanguínea, tempo operatório ou complicações pós-operatórias. Entretanto, contribuiu para uma redução na permanência hospitalar em comparação com a técnica convencional.

## Abstract (en)

**Objective:**

To investigate the outcomes of total knee arthroplasty (TKA) when assisted by a robotic arm compared with the conventional approach.

**Methods:**

We conducted a retrospective cohort study including 96 patients who underwent TKA, assigned to either the robotic-assisted (RA) group or a conventional technique (CT) groups. All surgeries were performed without the use of a tourniquet and included administration of intravenous tranexamic acid. Patients were matched based on sex, age, and preoperative hemoglobin (Hb) and hematocrit (Ht) values. Key outcomes evaluated were perioperative blood loss (assessed through changes in Hb and Ht), operative duration, length of hospital stay, and postoperative complications up to 6 months.

**Results:**

There were 34 patients from each group successfully matched for analysis. There were no significant differences between the groups regarding Hb or Ht reduction (RA: Hb −2.27 ± 1.21 g/dL, Ht −6.56 ± 3.43%; vs. CT: Hb −2.00 ± 1.07 g/dL, Ht −5.85 ± 3.26%; *p* > 0.05). The mean surgical time was also similar (RA: 108.9 ± 20.8 vs. CT: 111.8 ± 26.2 min; *p* = 0.905). Notably, patients in the RA group experienced a shorter hospitalization period (median: 2 vs. 2.5 days; OR = 0.12; 95% CI = 0.03–0.57; *p* = 0.008). Incidence of postoperative complications within 6 months did not differ significantly between groups.

**Conclusion:**

Robotic-assisted TKA was not associated with measurable improvements in blood loss, operative time, or postoperative complications. However, it contributed to a reduction in hospital stay compared with the conventional technique.

## Introdução

A artroplastia total do joelho (ATJ) é uma intervenção cirúrgica comum que visa aliviar a dor e restaurar a função em pacientes com osteoartrite grave ou outras condições articulares debilitantes. 1 Este procedimento envolve a substituição da articulação do joelho por uma prótese, removendo porções danificadas do fêmur, tíbia e cartilagem. 2 No entanto, a ATJ está associada a preocupações significativas quanto ao sangramento, o que pode levar a complicações pós-operatórias, aumentar o risco de transfusões e prolongar a recuperação do paciente. 2

Nas últimas décadas, a introdução da assistência de braço robótico revolucionou a precisão e a eficácia do procedimento, levantando questões importantes sobre seu impacto nos níveis de sangramento. 3 A ATJ convencional baseia-se em radiografias pré-operatórias, pontos de referência anatômicos intraoperatórios e guias de alinhamento manual, enquanto a ATJ com assistência robótica fornece uma reconstrução virtual tridimensional (3D) que descreve a anatomia específica de cada paciente. 4 5 Isso permite um planejamento mais preciso das ressecções ósseas e do posicionamento da prótese, melhorando assim a preservação do tecido e reduzindo a inflamação e a dor pós-operatória, 4 5 embora não tenham sido demonstradas diferenças significativas nos resultados funcionais de médio e longo prazo quando comparados com a técnica convencional. 6 7 8 9 10

Além disso, outras estratégias perioperatórias, como o uso do agente antifibrinolítico ácido tranexâmico, têm sido empregadas para reduzir a perda de sangue e a necessidade de transfusões. 11 Algumas metanálises mostraram que o ácido tranexâmico é seguro e reduz significativamente a perda sanguínea total e pós-operatória, bem como o número médio de transfusões por paciente, sem aumentar a taxa de complicações. 12 13 14

O uso de torniquete é tradicional na ATJ, baseada na crença de que podem reduzir o sangramento intraoperatório e melhora a cimentação da prótese. 15 No entanto, uma meta-análise publicada em 2021 indicou que o uso de torniquete pode não oferecer vantagens significativas nesse sentido e está associado a riscos, como danos aos tecidos moles, maior dor pós-operatória e maior tempo de internação hospitalar. Embora possa reduzir o tempo cirúrgico, esses potenciais efeitos adversos destacam a necessidade de reconsiderar seu uso rotineiro. 15

Até o momento, não há consenso sobre as taxas de sangramento na ATJ com assistência robótica, particularmente quando realizada em combinação com ácido tranexâmico intravenoso e sem o uso de torniquete. 16

A hipótese deste estudo é que a ATJ com assistência robótica resulta em menor sangramento perioperatório, menos complicações pós-operatórias e menor tempo cirúrgico quando comparada ao método cirúrgico convencional.

## Materiais e Métodos

### Aspectos Éticos

Este estudo foi realizado após aprovação do Comitê de Ética em Pesquisa Institucional, número CAAE 79863824.2.0000.5696, de acordo com a Resolução n° 466/2012 do Conselho Nacional de Saúde e a Declaração de Helsinki.

### Desenho do Estudo

Trata-se de um estudo observacional retrospectivo, baseado na análise de prontuários de pacientes submetidos à ATJ pela mesma equipe de cirurgia do joelho, entre julho de 2022 e 2023. Os pacientes foram divididos em dois grupos distintos: um em que a ATJ foi realizada com assistência robótica e outro com método convencional.

Os critérios de inclusão compreenderam todos os pacientes que realizaram ATJ sem uso de torniquete e com administração de ácido tranexâmico. Os critérios de exclusão foram ATJ de revisão, osteotomia corretiva concomitante, distúrbios de coagulação anteriores ou uso de medicação anticoagulante nos 7 dias anteriores à cirurgia.

## Coleta de Dados

Os seguintes dados foram coletados dos prontuários dos pacientes durante a internação: sexo, idade, peso, altura, distúrbios de coagulação prévios, data da cirurgia, tempo cirúrgico, assistência robótica, assim como uso de medicamentos anticoagulantes, ácido tranexâmico e torniquete. Também se coletou dados sobre complicações intraoperatórias, necessidade de procedimentos adicionais, níveis de Hb e Ht pré- e pós-operatórios de 24 horas, tempo de internação hospitalar e complicações pós-operatórias até o 6° mês, incluindo-se na última variável necessidade de transfusões, reoperação para drenagem de hematoma, artrofibrose, deiscência de ferida, infecção superficial e profunda, trombose venosa profunda (TVP), embolismo pulmonar (EP), infarto do miocárdio e óbito.

Todos os pacientes incluídos tiveram amostras de sangue coletadas por punção venosa periférica no pré-operatório e 24 horas após a cirurgia. O tempo cirúrgico de cada procedimento foi registrado pelas equipes de enfermagem e de anestesia. Todos os pacientes foram submetidos à raquianestesia e receberam 1 g de ácido tranexâmico por via intravenosa na indução. A abordagem parapatelar medial sem uso de torniquete foi a preferida.

Todos os implantes foram cimentados, sem substituição patelar e com a mesma instrumentação. No grupo com assistência robótica, utilizou-se o sistema de joelho ROSA (Zimmer Biomet). Nenhum dreno de sucção foi aplicado em nenhum caso.

A tromboprofilaxia consistiu em métodos mecânicos, com o uso de um dispositivo de compressão pneumática intermitente nas panturrilhas durante as primeiras 24 horas de pós-operatório, assim como meias elásticas de compressão durante os primeiros 30 dias. A profilaxia farmacológica incluiu 10 mg de rivaroxabana por via oral, iniciada 6 horas após o procedimento e mantida na mesma dose a cada 24 horas por 10 dias em todos os pacientes.

Os critérios de alta hospitalar incluíram estabilidade hemodinâmica, controle adequado da dor e capacidade de deambular de forma independente com um andador.

### Análise Estatística

Tanto Hb quanto Ht foram consideradas variáveis dependentes, enquanto idade, sexo, tempo cirúrgico, tempo de internação e complicações (inclusive óbito) foram considerados independentes. As variáveis categóricas foram expressas em frequência e porcentagem, enquanto as quantitativas foram avaliadas quanto à normalidade pelo teste de Shapiro-Wilk. A significância estatística das variáveis qualitativas foi avaliada pelo teste exato de Fisher, e as variáveis quantitativas foram analisadas pelo teste de Mann-Whitney ou pelo *t* de Student, conforme a distribuição dos dados.

Além disso, uma análise de regressão multivariada, ajustada por idade e sexo, foi realizada, utilizando o teste de Wald, para determinar o impacto da assistência robótica nas reduções de Hb e Ht, do tempo cirúrgico, da internação hospitalar e das taxas de complicações. Todas as análises foram realizadas no Stata/SE v.16.1 (StataCorp LLC), com o nível de significância estabelecido em *p* < 0,05.

## Resultados

**Fig. 1:**
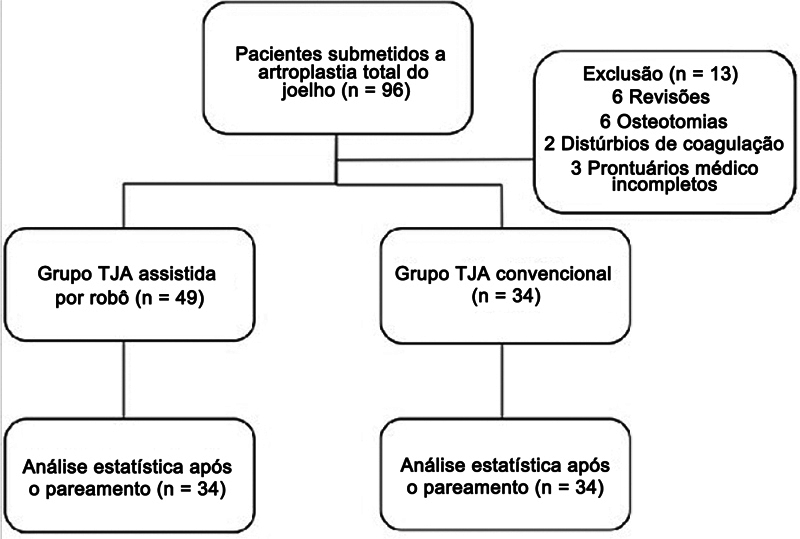
Fluxograma do estudo.

Durante o período do estudo, um total de 96 pacientes foi submetido à ATJ sem uso de torniquete e com administração de ácido tranexâmico. Do total, 85 pacientes foram incluídos no estudo; além disso, 6 foram excluídos devido à cirurgia de revisão, 2 devido à osteotomia associada e 2 devido a distúrbios de coagulação. Após a correspondência estatística dos grupos, o grupo de cirurgia com assistência robótica e o de cirurgia convencional compreenderam 34 pacientes cada. O fluxograma do estudo é apresentado na Fig. 1 .

A Tabela 1 apresenta as características basais dos pacientes. As médias de idade, a distribuição por sexo e os níveis basais de Hb e Ht foram semelhantes entre os grupos, sem diferenças estatisticamente significativas ( *p* > 0,05).

**Tabela 1:** Características basais dos pacientes

	Assistência robótica ( *n* = 34)	Convencional ( *n* = 34)	Valor de *p*
Idade (anos)	67,8 ± 9,7	68,2 ± 7,4	0,845 ^a^
Homens, n (%)	26 (53,1)	13 (38,2)	0,263 ^b^
Hb (g/dl)	13,56 ± 1,05	13,05 ± 1,31	0,079 ^a^
Ht (%)	40,32 ± 3,15	39,49 ± 4,19	0,358 ^a^
Tempo de cirurgia (min)	108,9 ± 20,8	111,79 ± 26,17	0,621 ^a^
Internação hospitalar (dias)	2 (1–3)	2 (2–3)	0,008 ^c^
Transfusão (n)	0	0	
Infecção superficial (n)	4 (11,8)	1 (2,9)	0,356 ^b^
Infecção profunda (n)	2 (5,6)	1 (2,9)	> 0,999 ^b^
Artrofibrose (n)	1 (2,9)	1 (2,9)	> 0,999 ^b^
Trombose (n)	0 (0,0)	1 (2,9)	> 0,999 ^b^
Embolismo (n)		0	
Ataque cardíaco (n)	1 (2,9)	0 (0,0)	> 0,999 ^b^
Morte (n)	0	0	

Abreviações: Hb, hemoglobina; Ht, hematócrito. a Teste t, b Qui-quadrado, c Teste de Mann-Whitney.

O tempo cirúrgico e o de internação também foram avaliados. Não houve diferença significativa no tempo cirúrgico entre os grupos ( *p* = 0,621). Entretanto, houve diferença significativa no tempo de internação, com menor tempo de permanência no grupo com assistência robótica ( *p* = 0,008), como apresentado na Tabela 1 .

Quanto às complicações cirúrgicas, os resultados não mostraram diferenças estatisticamente significativas entre os grupos em nenhuma das variáveis. Não foram identificados casos de EP, de necessidade de transfusão ou de óbitos ( Tabela 1 ).

Como mostrado na Tabela 2 , as diferenças nos níveis médios de Hb e Ht pré- e pós-operatórios (24 h) não foram estatisticamente significativas entre os grupos.

**Tabela 2:** Diferença média para Hb e Ht em pacientes submetidos a ATJ com assistência robótica ou convencional no período pré-operatório

Tempo	Assistência robótica Média (DP)	Convencional Média (DP)	Valor de *p*
Hemoglobina (mg/dl)			
24h	−2,27 (1,21)	−2,00 (1,07)	0,329
Hematócrito (%)			
24h	−6,56 (3,43)	−5,85 (3,26)	0,383

**Abreviações:** ATJ, artroplastia total do joelho; DP, desvio padrão; Hb, hemoglobina, Ht, hematócrito.

Na Tabela 3 , a análise de regressão multivariada ajustada para idade e sexo mostrou que a assistência robótica teve impacto significativo apenas na redução do tempo de internação hospitalar (OR = 0,12; IC 95% = 0,03–0,57; *p* = 0,008), sem influência significativa sobre outras variáveis (Hb, Ht, tempo cirúrgico e complicações).

**Tabela 3:** Efeito da assistência robótica na redução de Hb e Ht, dias de hospitalização, tempo de ATJ e complicações *

	OR	95% IC	Valor de P
Redução de Hb	1,83	0,67–4,99	0,236
Redução de Ht	0,91	0,65–1,23	0,594
Tempo de cirurgia	1,01	0,98 - 1,03	0,905
Tempo de internação hospitalar	0,12	0,03–0,57	0,008
Infecção superficial	0,23	0,01–4,03	0,316
Infecção profunda	2,19	0,08–60,1	0,642
Artrofibrose	0,78	0,04–15,0	0,868

**Abreviações:** ATJ, artroplastia total do joelho; DP, desvio padrão; Hb, hemoglobina, Ht, hematócrito; OR, odds ratio.

**Nota:** *Análise de regressão multivariada. Teste de Wald. Ajustada por idade e sexo.

## Discussão

Este estudo não demonstrou diferenças significativas na redução dos níveis de Hb e Ht após a cirurgia entre os grupos ( *p* > 0,05). Esse achado sugere que, na ausência de torniquete e com administração intravenosa de ácido tranexâmico, a ATJ com assistência robótica não apresenta vantagem clara quanto à perda sanguínea em comparação com a técnica convencional.

Esses resultados são consistentes com um estudo retrospectivo publicado em 2022, 16 que incluiu 486 pacientes e empregou torniquete e ácido tranexâmico. Eles também se alinham com uma revisão sistemática e meta-análise publicada em 2024, 17 que avaliou 12 estudos envolvendo 2.863 pacientes e analisou os requisitos de transfusão e de perda sanguínea em ATJ convencional versus com assistência robótica. No entanto, essa revisão não abordou o uso de ácido tranexâmico ou de torniquete nos estudos incluídos. 17

Nenhum dos pacientes do presente estudo necessitou de transfusão de sangue. Em contraste, um estudo de coorte retrospectivo de Khan et al., publicado em 2021, relatou seis casos de transfusão no grupo convencional (12%) e um no grupo com assistência robótica (2%), representando uma diferença estatisticamente significativa. 2 Nesse estudo, tanto o ácido tranexâmico quanto o torniquete foram utilizados em todos os pacientes. 2

O tempo cirúrgico também não diferiu significativamente entre os grupos no nosso estudo. A duração média foi de 108,8 ± 20,8 minutos no grupo com assistência robótica, em comparação a 111,79 ± 26,17 minutos no grupo convencional ( *p* = 0,484). Um estudo randomizado controlado relatou que a ATJ com assistência robótica foi associada a um tempo operatório mais longo em comparação com a abordagem convencional, 18 enquanto um estudo qualitativo descritivo sugeriu que a assistência robótica deve reduzir o tempo cirúrgico. 19 A tecnologia robótica permite simulações pré-operatórias, prevenindo erros no posicionamento da prótese e auxiliando a tomada de decisão de cirurgiões. 19 No entanto, tais procedimentos às vezes podem levar mais tempo, especialmente durante a curva de aprendizado inicial. 20 Em nosso estudo, as cirurgias com assistência robótica foram realizadas no 1° ano após a implementação do sistema em nossa instituição, mas não foi observada diferença no tempo cirúrgico.

Complicações pós-operatórias classicamente descritas em TJA não apresentaram diferenças significativas entre os grupos, 21 22 23 entre as quais estão necessidade de transfusão, reoperação para drenagem de hematoma, artrofibrose, deiscência de ferida, infecção superficial e profunda, TVP, EP, infarto do miocárdio e morte. Isso é consistente com um estudo de coorte retrospectivo publicado em 2022, analisando um grande banco de dados de ATJ, 24 que não encontrou associação entre a crescente adoção de ATJ com assistência robótica e taxas mais altas de complicações infecciosas ou não infecciosas.

Em relação às infecções, o estudo retrospectivo de Ofa et al., 25 publicado em 2020, comparou a ATJ com assistência robótica à convencional e não encontrou diferença significativa aos 90 dias de pós-operatório. As taxas de infecção foram de 0,62% no grupo convencional e 0,48% no grupo com assistência robótica. 25 Em nosso estudo, não foram observadas diferenças estatisticamente significativas, mas a taxa geral de infecção foi maior do que a relatada na literatura, atingindo 4% aos 6 meses.

Em relação à TVP, nosso estudo identificou um caso em cada grupo, sem significância estatística. Um estudo retrospectivo de Itou et al., publicado em 2023, 26 que utilizou tanto o torniquete quanto o ácido tranexâmico, também descobriu que a ATJ com assistência robótica não estava associada ao aumento do risco de TVP pós-operatória. Outra série de casos retrospectivos consecutivos sugeriu que, apesar de tempos operatórios mais longos, a cirurgia com assistência robótica pode ser mais segura, associada a menor incidência de TVP. 5 Isso pode ser atribuído ao menor trauma de partes moles e à prevenção de violação do canal femoral. 5

Além disso, um estudo caso-controle randomizado publicado em 2022 por Xu et al. 20 comparou os resultados clínicos e radiográficos precoces de ATJ com assistência robótica versus manual convencional com o uso de torniquete, não relatando diferenças significativas na incidência de TVP. Ambos os estudos enfatizaram o papel dos regimes anticoagulantes, como os inibidores do fator Xa, na redução do risco de TVP pós-operatória, o que é consistente com nosso protocolo de estudo. 18 20 27

Encontramos uma redução significativa na permanência hospitalar no grupo com assistência robótica (IC95%: 0,03–0,57, *p* = 0,008), indicando um potencial benefício dessa tecnologia para aprimorar a recuperação pós-operatória. Estudos anteriores que compararam a ATJ convencional à com assistência robótica também demonstraram hospitalizações mais curtas. 26 27 28 Por exemplo, um estudo de coorte retrospectivo publicado em 2024 relatou um tempo médio de permanência de 1,89 dias no grupo com assistência robótica, em comparação a 2,41 dias no grupo convencional. 27 Outros estudos retrospectivos publicados em 2018 e 2024 também relataram reduções significativas na duração da hospitalização em pacientes de ATJ com assistência robótica. 27 28

Em nosso estudo, os critérios de alta foram baseados na estabilidade hemodinâmica, na capacidade de deambular com andador e no controle adequado da dor. Vale ressaltar que os estudos comparativos revisados aqui também relataram o uso de ácido tranexâmico durante a cirurgia, mas não forneceram critérios específicos de alta. Além disso, em um estudo, 27 o torniquete pneumático foi usado, mas nunca inflado, enquanto outro 28 não especificou se um torniquete foi aplicado.

Este estudo apresenta algumas limitações. O mais importante sendo o seu desenho retrospectivo, que pode ter afetado a precisão dos resultados. Além disso, ser conduzido em um único centro limitou o tamanho da amostra. Por outro lado, essa abordagem minimizou o viés da variabilidade entre cirurgiões na técnica. Além disso, não foram coletados dados sobre desfechos funcionais ou escores de dor, o que impossibilitou uma comparação mais abrangente.

## Conclusão

Neste estudo, a escolha da abordagem cirúrgica—ATJ com assistência robótica versus convencional—não resultou em diferenças significativas nos níveis de hemoglobina ou hematócrito 24 horas após a cirurgia. Da mesma forma, o tempo operatório, a necessidade de transfusões de sangue e a incidência de complicações pós-operatórias foram comparáveis entre as duas técnicas.

Notavelmente, os pacientes submetidos à ATJ com assistência robótica tiveram uma permanência hospitalar significativamente mais curta, indicando uma vantagem potencial dessa tecnologia na promoção de uma recuperação pós-operatória mais rápida e alta precoce.
